# Association of Systemic Collagen Type IV Formation with Survival among Patients Undergoing Hemodialysis

**DOI:** 10.1371/journal.pone.0071050

**Published:** 2013-08-22

**Authors:** Diana J. Leeming, Morten A. Karsdal, Lars M. Rasmussen, Alexandra Scholze, Martin Tepel

**Affiliations:** 1 Nordic Bioscience, Herlev, Denmark; 2 Odense University Hospital, Department of Clinical Biochemistry and Pharmacology, Odense, Denmark; 3 Odense University Hospital, Department of Nephrology, Institute for Molecular Medicine, Cardiovascular and Renal Research, Institute of Clinical Research, University of Southern Denmark, Odense, Denmark; University of Campinas, Brazil

## Abstract

**Objective:**

The 7S domain of collagen type IV (P4NP_7S) assessed in plasma represents systemic collagen type IV formation. The objective of the study was to investigate the association of systemic collagen type IV formation with survival among patients undergoing hemodialysis.

**Methods:**

We performed an observational cohort study of 371 hemodialysis patients. Plasma P4NP_7S was analyzed using a specific enzyme-linked immunosorbent assay detecting the amino-terminal propeptide of type IV procollagen. Association between categories of plasma P4NP_7S concentrations and survival was initially assessed by Kaplan-Meier analysis, then in an adjusted Cox model.

**Results:**

For hemodialysis patients in the highest category of systemic collagen type IV formation, i.e. plasma P4NP_7S concentrations more than 775 pg/L, an increased risk for death was observed (highest P4NP_7S category vs all other categories, hazard ratio, 1.934; 95% confidence interval, 1.139 to 3.285). Survival analysis showed an increased risk of death in the highest P4NP_7S category compared to the other categories (Chi square 6.903; P = 0.032). Median survival was only 105 days in the highest P4NP_7S category whereas it was 629 days in the medium category, and 905 days in the lowest category. Multivariable-adjusted Cox regression showed increased odds for death with higher age and higher P4NP_7S categories. Systemic collagen type IV formation was associated with plasma concentrations of the collagen IV degradation product C4M (Spearman r = 0.764; P<0.0001) confirming extracellular matrix turnover.

**Conclusion:**

Among hemodialysis patients elevated systemic collagen type IV formation suggesting accelerating systemic fibrosis was associated with increased risk of death.

## Introduction

Excess mortality in patients with chronic kidney disease compared to the general population is still an important problem which needs to be addressed [1]–[3]. Using data from the European Renal Association–European Dialysis and Transplant Association registry, DeJager et al. reported that dialysis patients have a generally increased risk of death compared to the general population (overall all-cause mortality rate, 192 per 1000 person-years vs 12 per 1000 person-years) [4]. Recent investigations indicated that systemic fibrosis may contribute to excess mortality in patients with chronic kidney disease [5]. Increased inflammation, oxidative stress and increased levels of transforming growth factor-β are commonly observed in patients and animal models with chronic kidney disease [6]–[8]. In chronic kidney disease an increased inflammatory response triggers a fibrotic reaction of the connective tissue in several organs mainly characterized by an increased production of extracellular matrix components and mesenchymal cell proliferation, migration and accumulation [9]. Enhanced fibrosis in patients with chronic kidney disease is a systemic event. For example, key features of uremic cardiomyopathy include enhanced cardiac fibrosis, left ventricular hypertrophy, and systolic dysfunction [10]–[12].

Collagen type IV is primarily located in basement membranes of heart, lung, vessels, liver, and kidney tissue. Several studies have shown elevated expression of collagen type IV in the basement membrane from patients with chronic kidney disease [13]–[15]. Pathology studies indicate that the extent of collagen type IV deposition correlated with the severity of interstitial histological abnormalities [16]. Furthermore, recent clinical investigations indicate that increased urinary collagen type IV excretion is associated with progression of kidney function decline in diabetes mellitus type 2 and diabetes mellitus type 1 patients [17], [18].

During collagen turnover proteins are degraded which results in the release of unique and specific fragments into the circulation. The amino-terminal propeptide of type IV procollagen (P4NP) is an extension peptide of the procollagen type IV, which is cleaved off stoichiometrically during conversion from type IV procollagen to type IV collagen. After cleavage by proteases the fragment P4NP_7S is liberated into the systemic level and can be assessed in plasma [19]. The molecular weight of the P4NP_7S fragment is approximately 13,670 daltons. P4NP_7S represents systemic collagen type IV formation, including synthesis and deposition as well as altered degradation and elimination. Human plasma P4NP_7S concentrations have already been described as a marker for systemic fibrosis [13], [20], nevertheless using techniques that employ polyclonal antibodies in contrast to the present assay that uses a monoclonal antibody and thus is specific for a single epitope in the P4NP_7S domain. Furthermore, no data are available whether the extent of systemic fibrosis may be associated with mortality in hemodialysis patients. In the present study we investigated the association of systemic collagen type IV formation with survival among patients undergoing hemodialysis.

## Methods

### Ethics Statement

All research involving human participants was approved by the local ethics committee (Ethikkommission Free University Berlin, Reference numbers: ek.211-19, ek.Te2.02). Informed consent was obtained and all clinical investigation has been conducted according to the principles expressed in the Declaration of Helsinki. Written informed consent was obtained from all patients before entry into the study.

### Study population

To determine associations among plasma soluble 7S domain of collagen type IV (P4NP_7S) concentrations and mortality in hemodialysis patients we performed a cohort study in 371 hemodialysis patients which were followed up for 5 years. Our eligibility criteria were broad and included all adult patients on hemodialysis treatment due to end-stage chronic kidney disease stage 5 and presence of informed consent. Exclusion criteria were absence of informed consent. Data on time since initiation of dialysis at inclusion and duration of hemodialysis treatment per session were obtained. All patients were routinely dialyzed for 4 to 5 hours three times weekly using biocompatible synthetic polysulfone membranes with no dialyser reuse. None of the patients had hemodiafiltration. Blood flow rates were 250 to 300 mL/min, dialysate flow rates were 500 mL/min, and dialysate sodium ranged from 135 mmol/L to 145 mml/L. Anticoagulation during hemodialysis was performed using heparin. No patient had argatroban, danaparoid, nor citrate anticoagulation. A history of gadolinium exposure had not been recorded. Blood pressure was measured predialysis in patients in a recumbent position. Predialysis blood samples were taken at study entry. Blood was collected immediately before the start of the hemodialysis session.

Clinical and laboratory data included age, gender, medications (use of angiotensin-converting-enzyme inhibitors, ß-blockers, calcium channel blockers, and erythropoietin), body mass index (calculated as weight in kilograms divided by height in meters squared), systolic and diastolic blood pressure, serum albumin, serum urea, serum calcium, serum potassium, serum phosphorus, parathyroid hormone, cholesterol, and Low-Density-Lipoprotein cholesterol.

182 patients (49%) died during the follow up. The causes of death were classified as cardiovascular, infection, cancer, or unknown.

### ELISA protocols

Plasma P4NP_7S concentrations were measured using a specific ELISA targeting the 7S domain of type IV collagen as developed by our group [19]. Briefly, the monoclonal antibody, which was specifically raised against the synthetic peptide derived from a unique site in the 7S domain of collagen type IV, Biotin-CGG-PGEILGHVPG, was purified from collected culture supernatants using HiTrap affinity columns (GE Healthcare Life Science, Little Chalfont, Buckinghamshire, UK) and labeled with horseradish peroxidase (HRP) using Lightning-Link-HRP-Conjugation-Kit (Innova Biosciences, Babraham, Cambridge, UK), according to the manufacturer's instructions. The calibrators were dissolved in phosphate buffered saline (PBS) and used in the assay in this matrix. This antibody does not exhibit any cross reactivity with other collagen structures. The P4NP_7S competitive ELISA procedure was as follows: A 96-well streptavidin-coated enzyme-linked immunosorbent assay plate from Roche (Roche Diagnostics; Hvidovre; Denmark), was coated with the biotinylated specific P4NP_7S peptide against which the antibody had been raised (Biotin-CGG-PGEILGHVPG) dissolved in assay buffer (50 mmol/L 1,3,5-Tris(4-carboxyphenyl)benzene, pH 7.4), incubated for 30 min at 20°C in the dark and subsequently washed in washing buffer (20 mmol/L Tris, 50 mmol/L NaCl, pH 7.2). Thereafter 20 µl of peptide calibrator or sample were added to appropriate wells, followed by 100 µl of HRP-conjugated monoclonal antibody NB102-1E6 and the plate was incubated for 1 hour at 20°C and washed. Finally, 100 µL tetramethylbenzinidine (Kem-En-Tec Nordic; Taastrup; Denmark) was added, the plate was incubated for 15 min at 20°C, and in order to stop the reaction, 100 µl of stopping solution (1% H_2_SO_4_) was added and the plate was analyzed in the ELISA reader at 450 nm with 650 nm as the reference (Molecular Devices, SpectraMax M; Sunnyvale; CA; USA). A calibration curve was plotted using a 4-parametric mathematical fit model and the concentration of each sample was determined. The assay detection range was 8 to 500 pg/L, the intra-assay variation was 9.7%, and the inter-assay variation was 11.7%. Additional experiments confirmed that determinations using this ELISA did not show any interferences with biotin, immunoglobulins or lipid fractions. The molecular weight of the P4NP_7S fragment is approximately 13,670 daltons. It may not pass commonly used hemodialysis membranes. However, it is unknown whether smaller fragments may occur in vivo.

Similarly, plasma concentrations of the collagen type IV matrix metalloproteinase (MMP) degradation product C4M were measured using a specific ELISA targeting the MMP-2/-9 degraded type IV collagen. The monoclonal antibody used in our assay was developed to selectively recognize this MMP-2 and -9 cleavage site. The assay detection range was 1 to 100 pg/L, the intra-assay variation was 4.8%, and the inter-assay variation was 12.1%.

### Statistics

Continuous variables were expressed as median with interquartile range and compared with non-parametric Mann-Whitney test or non-parametric Kruskal-Wallis test and Dunn's multiple comparison post-hoc test where appropriate. Associations between variables were tested using non-parametric Spearman correlation. Categorical variables were expressed as proportions and compared with the Chi-square test. Time-to-event analyses were performed using the Kaplan-Meier method. Comparisons of survival curves were performed using the log-rank (Mantel-Cox) test. Data were initially stratified by decils, then they were stratified into categories with the lowest category being the lowest plasma P4NP_7S decil the medium category being all intermediate plasma P4NP_7S decils, and the highest category being the highest plasma P4NP_7S decil. Unadjusted and multivariable-adjusted survival analyses were performed using the proportional hazards regression model. Multivariable models were constructed with backward variable selection, using P<0.05 for variable retention. 46 patients (12%) underwent kidney transplantation during the follow up. These patients were censored on the day of transplantation.

All hypothesis tests were 2-sided, with statistical significance defined as having a P value of less than 0.05. Statistical analyses were conducted using GraphPad Prism 5.0 (GraphPad Software, San Diego, CA) or SPSS for windows (version 15; SPSS, Chicago, IL).

## Results

### Characteristics of cohort at baseline

A total of 371 hemodialysis patients (246 men and 125 women) with a median age of 67 years (IQR, 57 to 75 years), a median time since initiation of dialysis of 250 days (IQR, 31 to 1122 days), and a median dialysis dose (kt/V) of 1.0 (IQR, 0.9 to 1.2) entered into the study. The cause of chronic kidney disease was hypertensive nephrosclerosis in 125 cases (34%), diabetic nephropathy in 121 cases (32%), chronic glomerular nephritis in 30 cases (8%), polycystic kidney disease in 10 cases (3%) and other/unknown in 85 cases (23%). None of the patients had Goodpasture's syndrome, Alport-syndrome or ankylosing spondylitis.

Plasma P4NP_7S concentrations were determined as a quantitative marker for systemic collagen type IV formation in systemic fibrosis. Median plasma P4NP_7S concentrations were 387 pg/L (IQR, 293 to 594 pg/L). P4NP_7S concentrations were similar in men and women (388 pg/L; IQR, 311 to 600 pg/L; n = 246; vs. 387 pg/L; IQR, 267 to 575 pg/L; n = 125; P = 0.247). P4NP_7S concentrations were similar for all causes of chronic kidney disease (nephrosclerosis, 363 pg/L; IQR, 282 to 591 pg/L; diabetic nephropathy, 407 pg/L; IQR, 316 to 579 pg/L; chronic glomerular nephritis, 453 pg/L; IQR, 318 to 656 pg/L; polycystic kidney disease, 350 pg/L; IQR, 273 to 478 pg/L; other/unknown, 397 pg/L; IQR, 302 to 610 pg/L; P = 0.521). P4NP_7S concentrations were similar in subgroups of patients with time since initiation of dialysis less or more than 90 days (incident dialysis patients, 419 pg/L; IQR, 293 to 618 pg/L; n = 135; vs. prevalent dialysis patients 378 pg/L; IQR, 293 to 579 pg/L; n = 236; P = 0.340).

**Table 1** summarizes clinical and laboratory variables stratified according to categories of plasma P4NP_7S concentrations. The lowest category patients had a P4NP_7S plasma concentration less than 235 pg/L, the medium category had plasma P4NP_7S concentrations from 235 pg/L to 775 pg/L, and the highest category had plasma P4NP_7S concentrations higher than 775 pg/L. Hemodialysis patients with plasma P4NP_7S concentrations in the highest category had lower systolic (P = 0.013) and lower diastolic blood pressure (P = 0.016).

**Table 1:** Baseline clinical and biochemical characteristics of prevalent hemodialysis patients by categories of plasma concentrations of 7S domain of collagen type IV (P4NP_7S).^a^

Characteristic	Lowest category	Medium category	Highest category	P-value
Age (years)	65 (56 to 74)	67 (56 to 75)	67 (64 to 77)	0.342
Gender (% Female)	46%	32%	35%	0.234
Time since initiation of dialysis (days)	518 (31 to 1437)	245 (31 to 1142)	134 (31 to 790)	0.344
Diabetes mellitus (%)	43%	38%	41%	0.900
Current smoker (%)	27%	31%	19%	0.278
Weight (kg)	68 (62 to 82)	72 (63 to 80)	70 (58 to 80)	0.812
Body mass index^b^ (kg/m^2^)	24.1 (22.3 to 27.2)	24.6 (22.0 to 27.6)	24.9 (20.9 t0 27.0)	0.817
Systolic blood pressure (mmHg)	131 (115 to 147)	136 (117 to 150)	118 (97 to 143)	0.013
Diastolic blood pressure (mmHg)	71 (58 to 85)	70 (59 to 81)	63 (54 to 70)	0.016
Hemoglobin (mg/dL)	10.3 (8.6 to 11.7)	10.2 (9.0 to 11.7)	10.0 (9.2 to 11.0)	0.794
Leukocytes (10^9^/L)	8 (5 to 11)	8 (6 to 11)	8 (6 to 12)	0.641
Platelets (10^9^/L)	214 (140 to 290)	231 (179 to 294)	198 (136 to 257)	0.056
Serum albumin (g/L)	3.5 (2.9 to 3.9)	3.3 (2.9 to 3.7)	3.1 (2.5 to 3.8)	0.097
Urea (mg/dL)	87 (61 to 119)	70 (52 to 99)	68 (45 to 84)	0.010
Serum potassium (mmol/L)	4.6 (4.1 to 5.5)	4.7 (4.2 to 5.2)	4.5 (4.0 to 5.3)	0.535
Serum calcium (mmol/L)	2.21 (2.09 to 2.42)	2.21 (2.08 to 2.40)	2.21 (2.07 to 2.36)	0.700
Serum phosphorus (mg/dL)	1.52 (1.20 to 2.10)	1.60 (1.20 to 2.07)	1.59 (1.09 to 2.13)	0.850
Parathyroid hormone (ng/mL)	95 (33 to 296)	109 (42 to 233)	96 (52 to 191)	0.861
Serum cholesterol (mg/dL)	178 (134 to 222)	158 (135 to 193)	139 (117 to 170)	0.019
Low-Density-Lipoprotein-cholesterol (mg/dl)	112 (84 to 128)	97 (75 to 122)	87 (64 to 106)	0.044
High-sensitive C-Reactive-Protein (mg/dL)	2.0 (0.4 to 4.3)	2.7 (1.0 to 5.5)	3.0 (1.4 to 5.6)	0.119
Dialysis dose (ktV)	1.13 (0.97 to 1.30)	1.17 (1.04 to 1.33)	1.25 (1.10 to 1.39)	0.138
Angiotensin converting enzyme inhibitors (%)	30%	25%	35%	0.376
ß-Blockers (%)	54%	62%	46%	0.121
Calcium channel blockers (%)	14%	32%	27%	0.070
Erythropoietin therapy (%)	38%	51%	54%	0.295

a Continuous variables are given as medians and interquartile range. Between categories, comparisons were made using non-parametric Kruskal-Wallis test for continuous variables and using Chi square test for categorical variables.

b Body mass index was calculated as weight in kilograms divided by height in meters squared.

Hemodialysis patients with plasma P4NP_7S concentrations in the highest category also had lower urea (P = 0.010), a proxy for reduced dietary protein intake. Furthermore, serum cholesterol was significantly lower in the highest P4NP_7S category (P = 0.019) and Low-Density-Lipoprotein-cholesterol was significantly lower in the highest P4NP_7S category (P = 0.044). Reduced dietary protein intake and malnutrition is often observed in patients with chronic kidney disease [21]. On the other hand, high-sensitive C-Reactive Protein concentrations were not significantly different between the categories (P = 0.119). Time since initiation of dialysis appeared little longer in patients with the lowest category of plasma P4NP_7S concentrations, however, it should be noted that time since initiation of dialysis were not significantly different between the categories (P = 0.344). Furthermore, age, gender, dialysis dose, hemoglobin concentrations, serum calcium concentrations, serum phosphorous concentrations, and number of patients using angiotensin converting enzyme inhibitors, ß-blockers, calcium channel blockers, and erythropoietin were similar in the categories.

### Analysis of outcome

**Figure 1:**
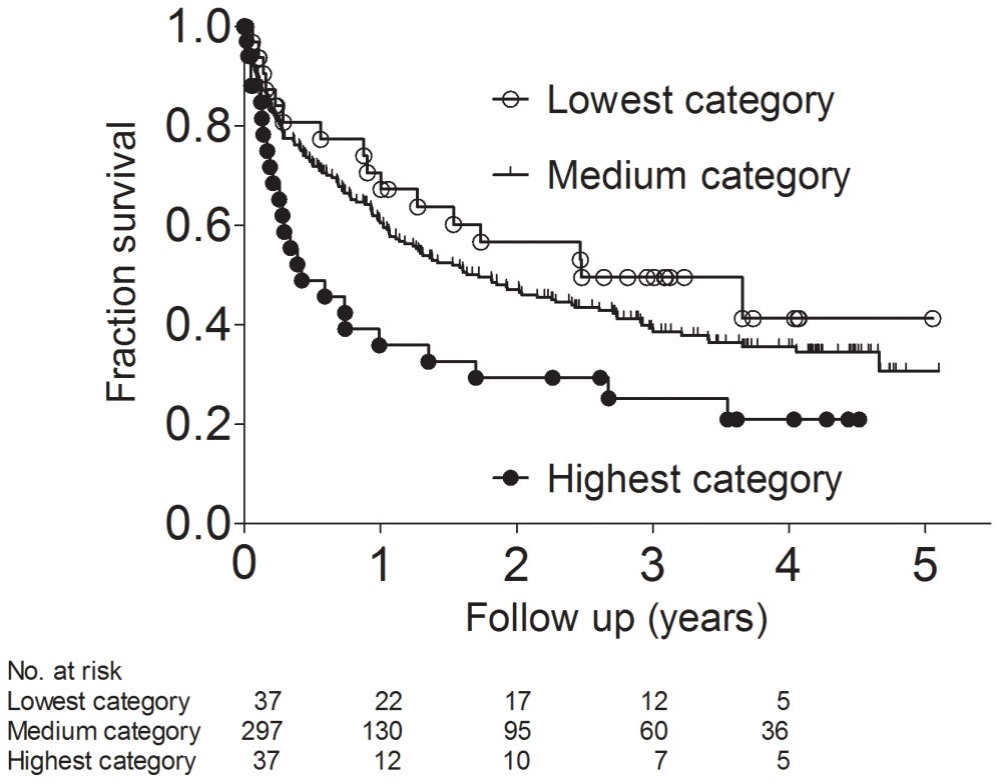
Kaplan-Meier survival curves for death in 371 hemodialysis patients. Patients were stratified according to lowest, medium, and highest categories of the 7S domain of collagen type IV (P4NP_7S) plasma concentration (Log rank test, chi square = 6.90; P = 0.032).

**Figure 2:**
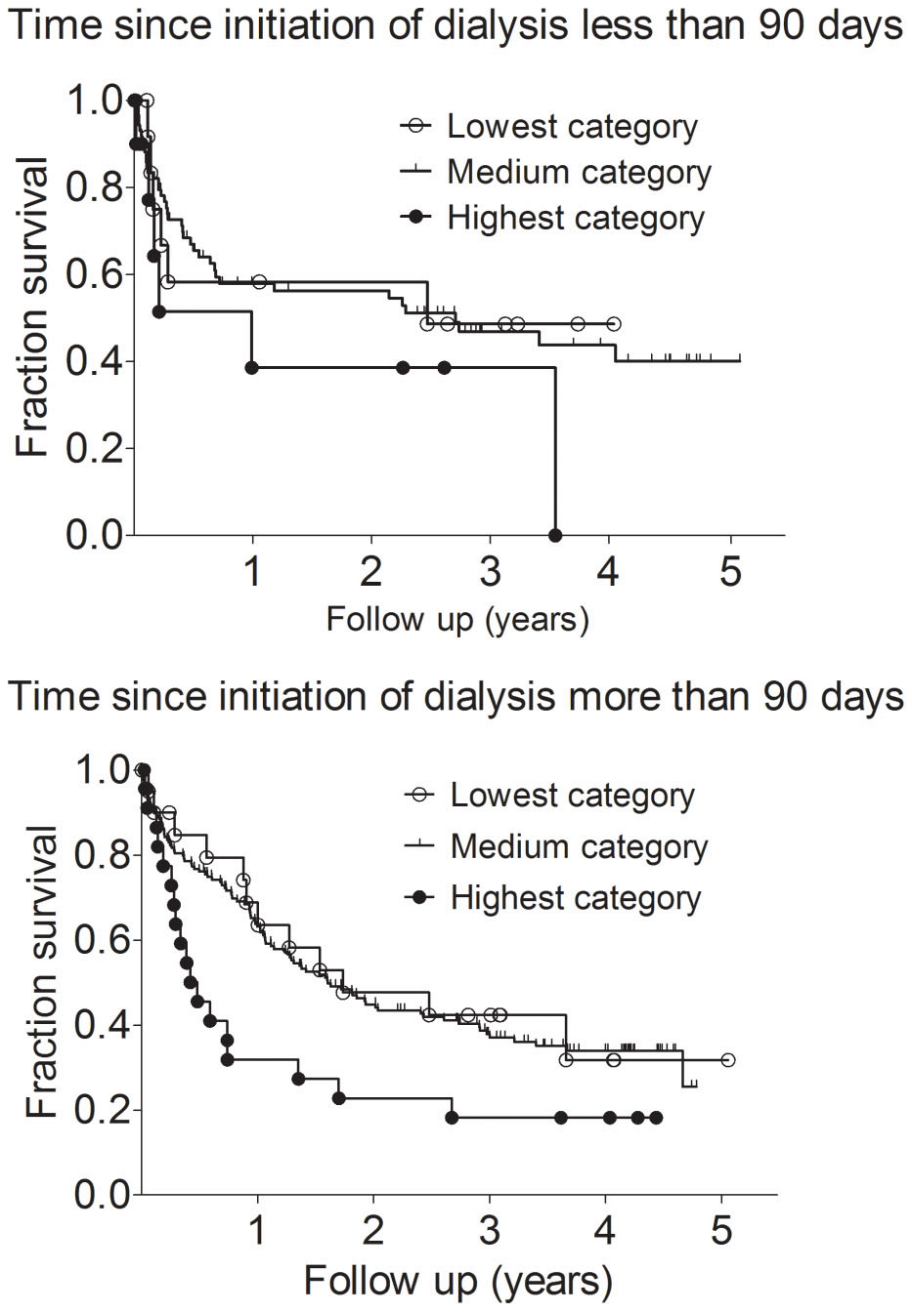
Kaplan-Meier survival curves for death in hemodialysis patients with time since initiation of dialysis less than 90 days (upper panel; log rank test, chi square = 1.70; P = 0.427) and in patients with time since initiation of dialysis more than 90 days (lower panel; log rank test, chi square = 6.63; P = 0.036). Patients were stratified according to lowest, medium, and highest categories of the 7S domain of collagen type IV (P4NP_7S) plasma concentration.

182 patients (49%) died during a follow up time of 5 years. Death occurred at a median of 201 days (IQR, 61 to 471 days) after study entry. The causes of death were cardiovascular diseases in 113 patients (62%), infections in 36 patients (20%), cancer in 22 patients (12%), and others/unknown in 11 (6%). In hemodialysis patients with the highest systemic collagen type IV formation an increased risk for death was observed (highest plasma P4NP_7S category vs all other categories, hazard ratio [HR], 1.934; 95% confidence interval [CI], 1.139 to 3.285, Mantel-Haenszel method). Further Kaplan-Meier analysis revealed significant survival differences between hemodialysis patients stratified into plasma P4NP_7S categories. Survival analysis showed an increased risk of death in the highest plasma P4NP_7S category compared to all other categories (Chi square 6.903; P = 0.032 by log-rank test). Median survival was only 105 days in the highest plasma P4NP_7S category, whereas it was 629 days in the medium category, and 905 days in the lowest plasma P4NP_7S category (**Figure 1**). In hemodialysis patients with the highest systemic collagen type IV formation the causes of death were cardiovascular diseases in 50%, infections in 34%, cancer in 8%, and others/unknown in 8%. Similar results were obtained in subgroups of patients with time since initiation of dialysis less or more than 90 days. In patients with time since initiation of dialysis less than 90 days the median survival was only 362 days in the highest plasma P4NP_7S category, whereas it was 901 days in the lowest plasma P4NP_7S category. In patients with time since initiation of dialysis more than 90 days the median survival was only 176 days in the highest plasma P4NP_7S category, whereas it was 634 days in the lowest plasma P4NP_7S category (**Figure 2**).

Results from univariable and multivariable-adjusted Cox regression are given in **Table 2**. Univariable Cox regression showed that plasma P4NP_7S category (P = 0.013), age (P<0.001) and systolic blood pressure (P = 0.032) were associated with mortality in hemodialysis patients, whereas gender (P = 0.696), diastolic blood pressure (P = 0.359), and urea (P = 0.176) were not associated with outcome.

**Table 2:** Univariable and multivariable Cox regression showing the odds for death in hemodialysis patients.

	UnivariableOdds Ratio (95% CI)	P	MultivariableOdds Ratio (95% CI)	P
Plasma P4NP_7S category	1.516 (1.091–2.106)	0.013	1.451 (1.050–2.004)	0.024
Age	1.058 (1.042–1.074)	<0.001	1.060 (1.044–1.076)	<0.001
Gender	1.068 (0.769–1.481)	0.696	1.068 (0.769–1.481)	0.696
Systolic blood pressure	0.991 (0.982–0.999)	0.032	0.988 (0.982–0.994)	<0.001
Diastolic blood pressure	0.993 (0.977–1.008)	0.359	0.993 (0.977–1.009)	0.377
Urea	1.002 (0.999–1.005)	0.176	1.002 (0.999–1.005)	0.173

Univariable survival analyses were performed using the proportional hazards regression model with all variable forced into the model. Multivariable-adjusted survival analyses were performed using the proportional hazards regression model with backward variable selection, using P<0.05 for variable retention.

Multivariable-adjusted Cox regression showed that the change in odds for death was 1.451 in higher plasma P4NP_7S category. As age is increased by one year the change in odds for death is 1.060. As systolic blood pressure is increased by one mmHg the change in odds for death is 0.988. In multivariable-adjusted Cox regression gender, diastolic blood pressure, and urea showed no effect.

### Assessment of collagen turnover

**Figure 3:**
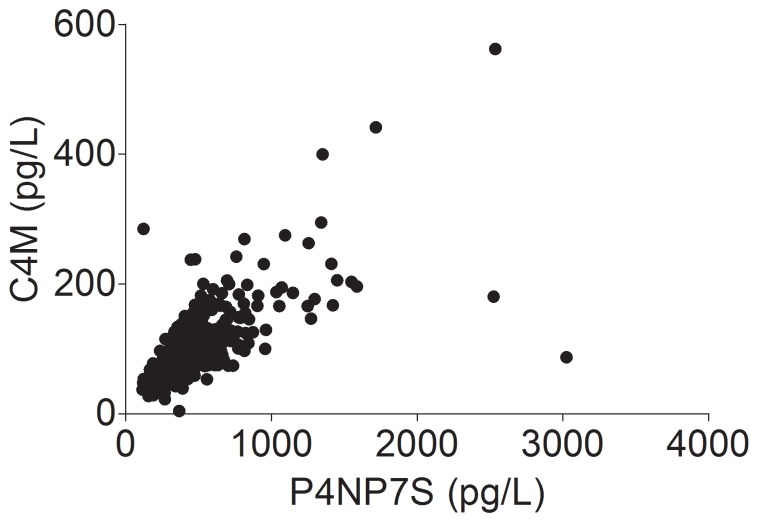
Association of 7S domain of collagen type IV (P4NP_7S) plasma concentrations with plasma concentrations of the collagen type IV degradation product C4M.

To assess extracellular matrix turnover in hemodialysis patients we also analyzed plasma concentrations of the collagen type IV degradation product C4M. We observed a significant association of P4NP_7S concentrations with C4M concentrations (Spearman r = 0.764; P<0.0001; **Figure 3**). Hemodialysis patients in the highest plasma P4NP_7S category had higher C4M concentrations (median, 180 pg/L; IQR, 147 to 218 pg/L) compared to the medium category (median, 90 pg/L; IQT 72 to 118 pg/L) and compared to the lowest category (median, 60 pg/L; IQR, 47 to 70 pg/L; P<0.0001). These findings suggest characteristic extracellular matrix turnover in hemodialysis patients.

## Discussion

The present study indicates that hemodialysis patients with high systemic collagen type IV formation had an increased risk for death. After multivariable adjustment higher plasma P4NP_7S concentrations and higher age increased the odds for death in hemodialysis patients. The demonstration of a significant effect of systemic collagen type IV formation on dialysis survival is novel, challenging conventional management of hemodialysis patients

Several biological mechanisms provide a plausible explanation for the association between high plasma P4NP_7S concentrations and death in hemodialysis patients. Plasma P4NP_7S concentrations are a quantitative marker for systemic collagen type IV formation in systemic fibrosis. Chronic tissue injury with activated fibrogenesis results in disruption of tissue architecture, organ dysfunction and finally organ failure and death. A universal feature of fibrosis is the complex interplay between the inflammatory-epithelial-myofibroblast and extracellular matrix components [22]. Increased oxidative stress in hemodialysis patients establishes pathological repair leading to accumulation of permanent fibrotic scar tissue at the site of injury. In addition to increased oxidative stress, accumulation of several uremic toxins, including indoxyl sulfate, can contribute to increased systemic fibrosis in chronic kidney disease [6], [21], [23]. Increased fibrosis is mainly supported by epithelial to mesenchymal transition which describes the transdifferentiation of an epithelial cell to a cell with myofibroblast-like features. Endothelial to mesenchymal transition has been described as a potential source of activated myofibroblasts causing fibrosis in chronic kidney disease [24], [25]. This fibrosis is characterized by the excessive accumulation of extracellular matrix components including collagens [24]. Increased glycation and oxidation of collagen type IV together with increased accumulation of extracellular matrix components has been reported in chronic kidney disease. These modifications of collagen type IV lead to the detachment and loss of functional cells [26]. Our present study is the first showing that hemodialysis patients with enhanced systemic fibrosis characterized by high plasma P4NP_7S concentrations had an increased risk for death. Earlier studies in both healthy and elderly individuals with cardiovascular disease had also shown that plasma markers of collagen turnover are significantly associated with myocardial infarction, heart failure, and death [27].

Enhanced systemic fibrosis presenting as uremic cardiomyopathy is a characteristic feature in hemodialysis patients [28]. As reviewed by Gross & Ritz, the collagen content of the cardiac interstitium has important functional consequences in uremic cardiomyopathy. Cardiac fibrosis is an important determinant for left ventricular compliance, systolic stress-strain relationship and local inhomogenities of electrical resistance [5]. Murakami et al. demonstrated elevated collagen type IV transcripts in the infarct zone of rat myocardial infarction, suggesting that type IV collagen contributes to the pathological response after myocardial injury [29]. In line with these observations, Herzog et al. reported poor long-term survival after acute myocardial infarction among hemodialysis patients [30]. According to our present study the identification of hemodialysis patients with high plasma P4NP_7S concentrations may help to uncover patients with increased risk for death due to systemic fibrosis. Such measurements may be advantageous with the advent of novel therapeutic strategies for systemic fibrosis [31], [32].

Furthermore, enhanced vascular fibrosis may also contribute to the observed increased risk for death with high systemic collagen type IV formation. Both, clinical and experimental studies indicated structural changes in the vasculature associated with chronic kidney disease [33], [34]. Accordingly, several authors have shown increased vascular stiffness in hemodialysis patients [35], [36].

It should be noted that P4NP_7S concentrations were similar in subgroups of patients with time since initiation of dialysis less than 90 days (incident dialysis patients) or more than 90 days (prevalent dialysis patients). Time since initiation of dialysis was not significantly different between the categories of plasma P4NP_7S concentrations. Furthermore, analyses of incident and prevalent subgroups of hemodialysis patients showed similar results, i.e. for both incident and prevalent subgroups, patients in the highest plasma P4NP_7S category showed higher mortality compared to patients in the lowest plasma P4NP_7S category. However, our data may indicate that hemodialysis may accelerate systemic fibrosis. Comparing survival times in the highest plasma P4NP_7S category showed that in patients with time since initiation of dialysis less than 90 days the median survival was 362 days, whereas in patients with time since initiation of dialysis more than 90 days the median survival was only 176 days.

Taken together, among hemodialysis patients elevated systemic collagen type IV formation was significantly associated with increased risk of death. This finding has implications for hemodialysis patients with regard to the mechanisms underlying elevated mortality risks and in the identification of systemic fibrosis in these patients. Determination of plasma P4NP_7S concentrations may help to uncover patients with increased risk for systemic fibrosis and death. In addition, because end-stage renal disease may be viewed as an in vivo model of accelerated systemic fibrosis, this study may have relevance for other renal and non-renal populations characterized by tissue fibrosis.
